# Integration of Waiting Room “Know Your Rights” Education into Medical Care of Immigrant Patients in a Federally Qualified Health Center: A Case Study

**DOI:** 10.1089/heq.2020.0145

**Published:** 2022-01-17

**Authors:** Andreé Franco-Vásquez, Stephanie Lemus, Kevin Castillo, Martin Isaac, Altaf Saadi

**Affiliations:** ^1^ David Geffen School of Medicine at University of California, Los Angeles, California, USA.; ^2^ Clínica Monseñor Oscar A. Romero, Los Angeles, California, USA.; ^3^ K-Cast Bookkeeping, Los Angeles, California, USA.; ^4^ Kheir Clinic, Los Angeles, California, USA.; ^5^ Department of Neurology, Massachusetts General Hospital, Harvard Medical School, Boston, Massachusetts, USA.

**Keywords:** immigrant health, legal rights education, medical legal collaboration, health disparities

## Abstract

**Background:** Federally qualified health centers (FQHCs) are implementing innovative interventions to address heightened anxieties of immigrant patients amid changing immigration policies.

**Purpose:** To describe the integration of “Know Your Rights” legal rights education in clinic waiting rooms of an FQHC in Los Angeles, California.

**Methods:** This is a qualitative study using key informant interviews, direct field observations, and document review.

**Results:** Collaboration with community health workers and local immigrant-serving community-based and legal organizations was key to intervention design and implementation.

**Conclusion:** Integrating legal education into medical care is one action health centers can take to support immigrant patients, address their complex realities, and optimize patients.

## Introduction

Among the >28 million patients who receive health care services from federally qualified health centers (FQHCs)^1^ regardless of their ability to pay are immigrant patients who face unique barriers to care. Undocumented immigrants are a subset of this population who are particularly vulnerable. They disproportionately work in hazardous low-paying jobs that do not offer employment-based health insurance and remain ineligible for health insurance despite expansions made under the Affordable Care Act.^2–10^

Undocumented immigrants also face sociopolitical stressors such as immigration enforcement, restrictive immigration policies, and anti-immigrant rhetoric at the federal,^4^ state,^5–9^ or local levels^10–12^ that further contribute to negative health outcomes. Fear of discovery and deportation dissuades use of needed health services,^3^ alongside being associated with poor mental health, worsening cardiovascular risk factors, lower self-reported general health, low birth weights among immigrant mothers, and reduced use of needed health care services.^13–15^ The health impacts of these sociopolitical stressors transcend legal status, impacting immigrants with authorization, U.S. citizens in mixed immigration-status households, and those belonging to the same ethnic/minority communities due to perceived illegality.^7,8,11,13^

To address immigrant patients' social needs, health systems have adopted “immigration-informed” interventions, including medical–legal partnerships that embed legal services into health care settings.^16–19^ Legally oriented interventions have most commonly focused on providing patients with one-on-one legal advice or assistance through legal personnel.^16,17^ These efforts expanded in the aftermath of the 2016 U.S. presidential election due to an increasingly anti-immigrant policy climate and heightened patient anxiety.^16,18,20–22^ Consequently, health systems implemented new interventions to support their immigrant patients, among them the integration of legal rights education within clinical care.^21^ Yet this has not been described in the medical or public health literature. This observational qualitative case study describes the implementation of “Know Your Rights” (KYR) legal rights education in clinic waiting rooms of Clínica Monseñor Oscar A. Romero (Clínica Romero), an FQHC in Los Angeles, California.

## Methods

We conducted key informant interviews, direct field observations, and document review from May to July 2018 using a descriptive qualitative case study approach. Clínica Romero was identified as part of a larger study assessing health care interventions aimed at mitigating immigration-related stressors for patients and providers.^21^

Key informants were staff members of Clínica Romero's Community Outreach and Patient Services Department (COPSD; *n*=4) who led implementation efforts for this intervention. Four field visits were conducted to observe KYR presentations (*n*=2) and UndocuHealth Youth Program training sessions for the KYR presentations (*n*=2). The UndocuHealth Youth Program was started in 2017 to foster community engagement and leadership skills development among local undocumented high school students while providing them a paid work opportunity.

**FIG. 1.**
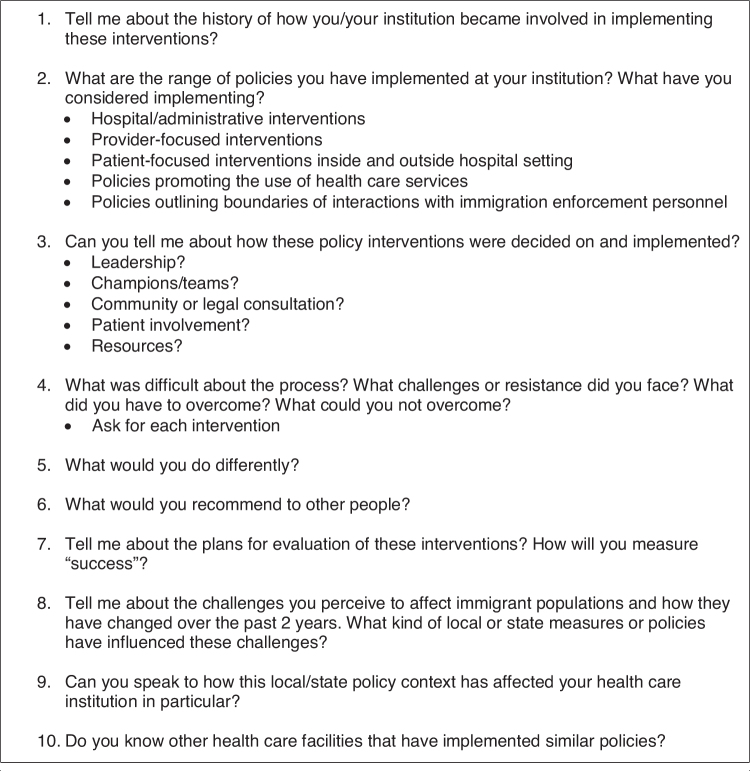
Semistructured interview guide.

Semistructured interviews (Fig. 1) with four key informants were conducted. Observations, field notes, and interview notes were taken on site and typed within 24 h, including reflective journaling. Authors A.S. and A.F.-V. conducted descriptive and content analyses iteratively and through consensus. This study was approved by the University of California, Los Angeles Institutional Review Board.

### Case study setting

Clínica Romero's two clinic locations serve 12,000 predominantly low-income foreign-born Latinos.^23^ Self-paying and uninsured patients comprise 43% of the patient population.^23^ The FQHC has 13 clinical providers, 2 behavioral health providers, 5 dentists, and 2 pharmacists.^24^ The FQHC's COPSD, Community Health Workers (CHWs, or *Promotores de Salud* in Spanish), and local immigrant-serving community-based organizations (CBO's) collaborate to improve civic engagement, capacity building, cultivation of resident leaders, and ensure enrollment in health programs.

## Results

### Designing and integrating the “Know Your Rights” intervention

To address patients' immigration-related concerns, the COPSD collaborated with CHWs and local immigrant-serving CBOs to design and implement a KYR legal education intervention (Table 1). Key informants described being motivated to address immigration-related concerns “to dismantle the social determinants of health” and to facilitate patient trust (“if [patients] don't trust [us], they're not going to come”) (see Table 2 for interview and field observation themes).

**Table 1.** Legal Education Topics Addressed in Waiting Room Know Your Rights Sessions

Topic	Components addressed	Sample wording
Legal rights regardless of immigration status	1. The right to remain silent 2. The right to speak to a lawyer 3. The right to make a phone call	“These rights are yours, regardless of your immigration status, whether you are citizens, residents, or undocumented.”
Response to law enforcement, including immigration enforcement	1. Do not open door 2. Ask for warrant to be slipped under the door 3. Determine whether warrant is valid 4. If invalid warrant: individual should take a picture and slip back to officers 5. If valid warrant: take a picture of warrant and cooperate with officers	“Now, if a police officer or an immigration agent knocks on the door, don't open the door and ask to get the warrant slipped under the door.”
Identification of valid warrants	1. Must come from a court 2. Must be signed by a judge 3. Address must match the address of the individual's residence 4. Must be handed to an individual before its expiration date	“A valid warrant says the word ‘court’ because it comes from a court. You have to make sure that the address of your house is the same as the one in the warrant. You also have to make sure on the expiration date. And the last and most important thing is that it has to be signed by a judge.”

**Table 2.** Themes and Descriptions

Themes from key informant interviews (n=4)	
Theme	Key informant quote
Addressing immigration concerns as a social determinant of health	“Because a lot of folks have so many issues. One of them being immigration, homelessness, food security. You name it. It's across the board. If you're not tackling those issues, if you're not opening up the door and putting out a table and chairs and saying, ‘Okay, let's talk about it,’ they're not going to.”
	“We wanted to show true commitment… [to] dismantle the social determinants of health and create systematic change.”
Addressing immigration concerns to demonstrate commitment and facilitate trust	“Our patients and our communities always deserve consistency and commitment… and building trust comes with a lot of commitment and a lot of consistency.”
	“It has to deal a lot with trust at the end of the day, because if they don't trust [us], they're not going to come.”
Patient and community empowerment	“We have to think for and by our patients, right? So we did a small assessment as to what the immediate needs were. And one of them was they didn't know their rights… so the things that we did were empower. We have developed groups of patients and community at large to not only know their rights, but also go and say their rights and teach others so it becomes a ripple effect.”
	“Empowering a person is giving them tools. It's the same way we teach cancer education. We're not going to say, ‘By the way you have 1 in 4 chances of getting cancer and you might die.’ No. We give them the problem, we give them the solution, and then give them the tools.”
Developing and strengthening clinic–legal partnerships	“We developed a public, private partnership that could assist the population that we were serving or that we are serving.”
	“We wanted to make sure that they had every tool needed for our efforts to be successful. We developed a list of private public partnerships. We had conversations with them. It was a lot of leg work, but it was well worth it. When we would go out and finally do any sort of trainings in our community, it was done correctly. We wanted to make sure that it was done with a lot of forethought in mind.”

Themes from field observations (n=4)	
Theme	Narrative description
Fostering patient trust throughout presentation	• KYR presentation information was offered in a nonjudgmental manner with offer for navigation support throughout the presentation (e.g., calling lawyer contact information, additional review of KYR cards or valid warrant examples). • KYR presentations were viewed as a method to connect with patients and strengthen trust in the clinic. Each interaction viewed as an opportunity to build trust. • Patients were invited to ask questions both in group setting during the presentation and in one-on-one after presentation was completed accommodating for different patient preference.
Informal but community-engaged evaluation process	• Presenters kept a general list of questions patients asked and tried to incorporate them into subsequent presentations so there was an iterative process in improving these presentations • No formal evaluation of patient-level response or satisfaction but *promotores* were in constant contact with patients and community members and brought feedback to COPSD team during weekly meetings • UndocuHealth youth program participants, who were members of the community, provided feedback on length and content of presentations
Observed patient responses to KYR presentations	• Many initially appeared indifferent but became more engaged when certain topics were discussed and resources or examples provided (e.g., lawyer contact information, KYR cards, valid warrant examples) • Most approached facilitator with individual questions after presentation concluded rather than asking questions in the group • Some only took the material or asked for materials and walked out of the waiting room without listening to the entire presentation. • Audience appeared more attentive when presentations were provided by staff rather than the youth • Patients expressed concern and fear of sharing personal information on sign-up sheets and referrals due to concerns of immigration status discovery
Observed barriers to effective presentations	• Noisy surroundings competed for patient's attention (e.g., staff calling patients through a loudspeaker, TV playing in the background in the waiting room) • Concerns of privacy Decreased waiting times interfered with full or effective presentations

COPSD, Community Outreach and Patient Services Department; KYR, “Know Your Rights.”

KYR presentations include discussion of legal rights, response to potential interactions with law enforcement, and identification of valid warrants (Table 1). The director of the COPSD used a “train the trainers” model^25^ to train COPSD staff as presentation facilitators. This training was supplemented by webinars, presentations, and conferences given by immigrant-serving organizations such as the National Immigration Law Center and Asian Americans Advancing Justice. COPSD staff subsequently trained other presentation facilitators: CHWs and UndocuHealth Youth Program participants.

In January 2017, Clínica Romero began providing 5-to-10-min daily KYR presentations in the waiting room. Information provided during the session was similar to KYR advice offered by community-based and legal organizations. Sessions were introduced in a neutral tone and information was framed as relevant to everyone, regardless of immigration status. Facilitators emphasized applicability of content to any law enforcement interactions, including Immigration and Customs Enforcement.

**FIG. 2.**
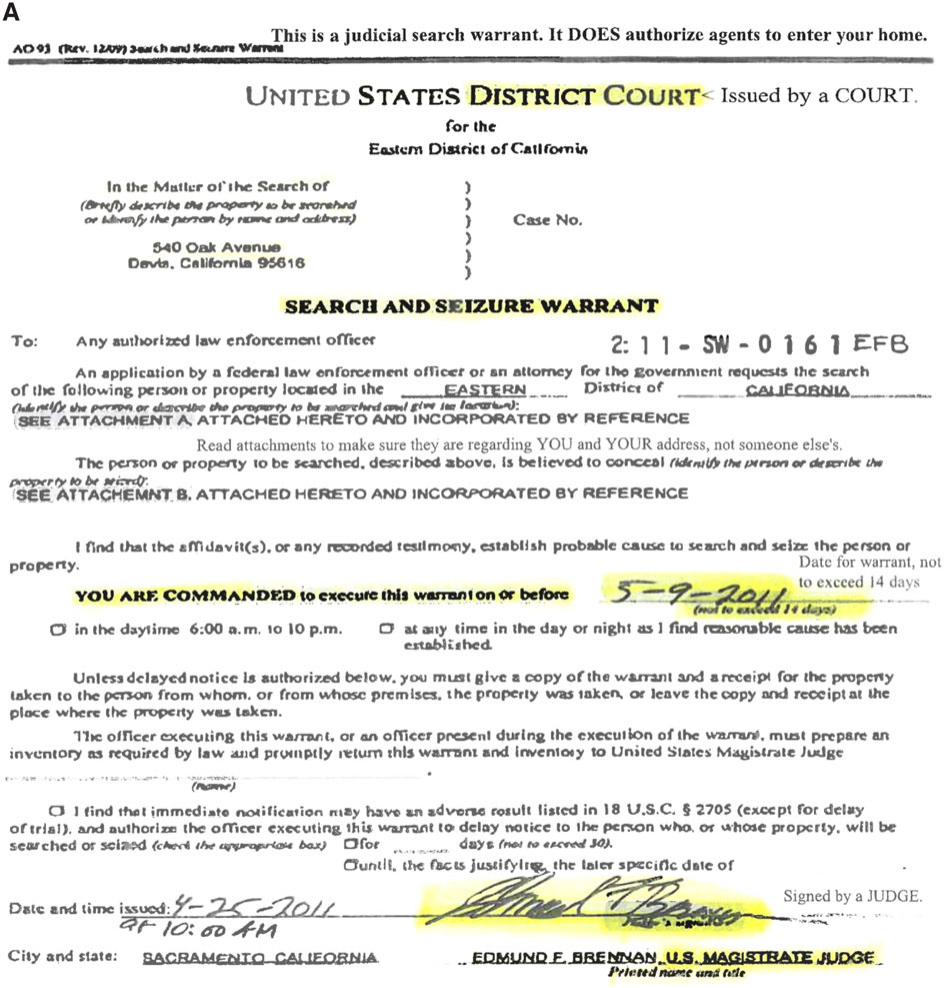

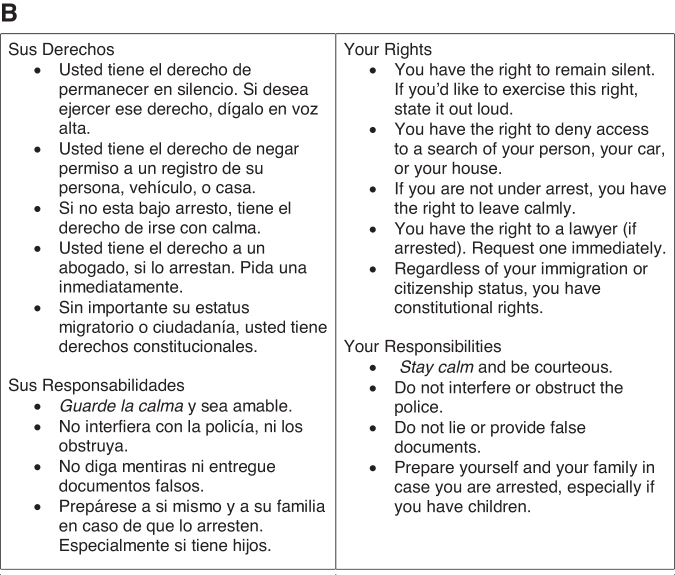
Know your rights presentation materials. **(A)** Sample of a valid search warrant. **(B)** Summary of individual rights and responsibilities in interactions with law enforcement.

**FIG. 3.**
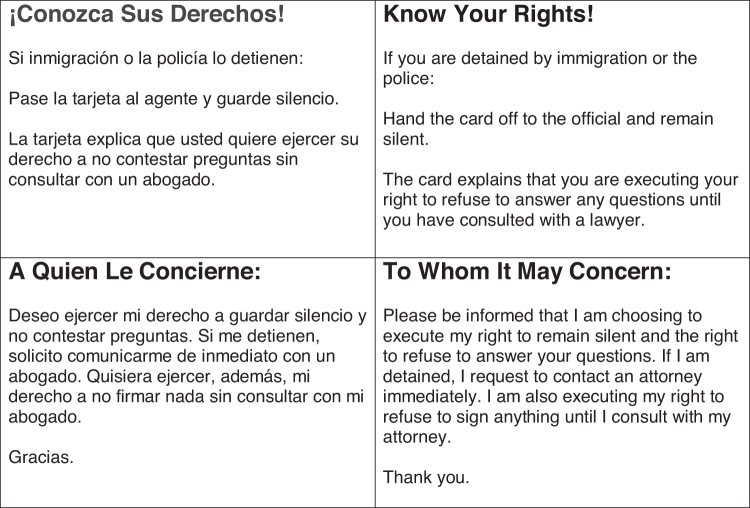
Printable bilingual Know Your Rights cards.

Facilitators used materials (Fig. 2) such as visual aids during presentations that were provided for patients to refer to and take home. These supplemental materials include a sample warrant (Fig. 2A), KYR brochure containing a summary of presentation content (Fig. 2B), KYR card (Fig. 3), and a local legal resource guide. Clínica Romero developed partnerships with legal organizations listed on their resource guide to provide patients a free initial consultation at these organizations. The facilitators offered community resource navigation support.

Waiting room KYR presentations were conducted in Spanish because >90% of the clinic's patients are Spanish-speaking only. Table 3 provides the number of patients seen from 2017 to 2020 (with a range of 2–10 patients in a waiting room at any given time). Facilitators encouraged questions throughout, and stayed in or around the waiting room for an additional 5–10 min after the formal presentation ended to address individual questions, share additional resources, and offer help with connecting to a lawyer if needed.

**Table 3.** Know Your Rights Education Presentation Outreach

	2017	2018	2019	2020
Total individuals reached^a^	15,824	5386	5340	13,974

^a^ Includes waiting room and community event KYR presentations.

### Program sustainability and evaluation

To ensure sustainability, the execution of the KYR presentations was integrated into the UndocuHealth Youth Program and the COPSD staff position responsibilities. CHWs also incorporated these presentations into their health education programming around conditions such as diabetes, hypertension, and mental health, in schools, churches, and other community venues. Weekly meetings with the CHW team ensured that feedback was exchanged between both teams to adapt these presentations as needed. Given staffing and funding limitations, formal evaluation of this intervention has yet to be conducted but is forthcoming.

### Program challenges

The waiting room setting posed various challenges for effective presentations, although providing a captivated audience. First, the noisiness of the waiting room sometimes distracted patients or overcame the facilitator's voice. To overcome this challenge, the team piloted conducting one-on-one presentations with patients within the waiting room. Another potential approach is turning off or lowering the volume of the waiting room television while presentations are occurring to minimize distractions. Second, the clinic noted variation in patient attendance with changing immigration policies, including ongoing public charge discussions^22^; this made concurrent presentations in other community spaces such as schools and churches vital to reaching and empowering the most patients possible rather than relying on clinic waiting rooms alone. Community events were conducted in English and Spanish to address language needs of attendees. Third, there was a tension between clinic goals: on one hand, decreasing waiting room times, and on the other, ensuring adequate time to provide legal information and resources. Having front desk staff provide patients with KYR materials at check-out could increase dissemination of information regardless of patient wait time. Fourth, there remain challenges with funding for this intervention and incorporating needs of different immigrant populations as African and Asian immigrants become members of the clinic population.

The COVID-19 pandemic has posed the ultimate challenge such as staff presence, in-person interactions, and waiting room time are minimized. The FQHC paused waiting room KYR presentations initially but later integrated them within COVID-19 response efforts (i.e., CHWs offering one-on-one KYR legal education to individuals receiving their COVID-19 testing or vaccinations).

### Future directions

Future study should assess patient perspectives of these interventions to most effectively address their concerns and promote trust in health care systems. This is particularly urgent during the COVID-19 pandemic when vaccination of all communities, particularly disproportionately affected immigrant communities, is needed for effective public health. Furthermore, FQHCs are particularly suited for these efforts given their position as a primary source of medical care for uninsured immigrants, trusted relationships, locations in the community, and enabling services such as language access; next steps could identify generalizability of this intervention to other health care facility types.

## Conclusion

FQHCs are uniquely positioned to serve as places of innovation to address the needs of communities they serve.^26^ This case study describes an innovative integration of KYR legal rights presentations into the clinical care of immigrant patients, developed in partnership with local community and legal organizations. It reflects a national trend of health care facilities addressing patient legal needs to improve health outcomes.^18^ In the setting of the COVID-19 pandemic, integrating provision of patient legal rights education into pandemic response efforts represents a critical strategy in addressing fears that may hinder testing or vaccination efforts. This case study underscores that there are many creative ways immigration-related fears can be addressed in the health care context to optimize equitable patient care for immigrants and their families.

## Abbreviations Used

CBO's: community-based organizations
COPSD: Community Outreach and Patient Services Department
FQHC: federally qualified health centers
KYR: “Know Your Rights”

## References

[1] National Association of Community Health Centers (NACHC). Bethesda, MD: America's Health Centers Fact Sheet. Available at: https://www.nachc.org/research-and-data/research-fact-sheets-and-infographics/americas-health-centers-2021-snapshot/. Accessed October 28, 2021.

[2] Passel JS, Cohn D. Unauthorized Immigrant Population: National and State Trends, 2010. Pew Research Center's Hispanic Trends Project. 2011. Available at http://www.pewhispanic.org/2011/02/01/unauthorized-immigrant-population-brnational-and-state-trends-2010 Accessed March 6, 2018.

[3] Hacker K, Anies M, Folb BL, Zallman L. Barriers to health care for undocumented immigrants: a literature review. Risk Manag Healthc Policy. 2015;8:175–183. 26586971 10.2147/RMHP.S70173 PMC4634824

[4] Fleming PJ, Lopez WD, Mesa H, et al. A qualitative study on the impact of the 2016 US election on the health of immigrant families in Southeast Michigan. BMC Public Health. 2019;19:947. 31307435 10.1186/s12889-019-7290-3 PMC6631662

[5] Stanhope KK, Hogue CR, Suglia SF, et al. Restrictive sub-federal immigration policy climates and very preterm birth risk among US-born and foreign-born Hispanic mothers in the United States, 2005–2016. Health Place. 2019;60:102209. 31550633 10.1016/j.healthplace.2019.102209

[6] Kline N. Pathogenic policy: immigrant policing, fear, and parallel medical systems in the US South. Med Anthropol. 2017;36:396–410. 27849361 10.1080/01459740.2016.1259621

[7] White K, Yeager VA, Menachemi N, et al. Impact of Alabama's Immigration Law on access to health care among Latina immigrants and children: implications for National Reform. Am J Public Health. 2014;104:397–405. 24432880 10.2105/AJPH.2013.301560 PMC3953801

[8] White K, Blackburn J, Manzella B, et al. Changes in use of county public health services following implementation of Alabama's immigration law. J Health Care Poor Underserved. 2014;25:1844–1852. 25418247 10.1353/hpu.2014.0194

[9] Wallace SP, Young M-EDT, Rodríguez MA, et al. A social determinants framework identifying state-level immigrant policies and their influence on health. SSM Popul Health. 2019;7:100316. 10.1016/j.ssmph.2018.10.016 PMC6293030 30581960

[10] Perreira KM, Pedroza JM. Policies of exclusion: Implications for the health of immigrants and their children. Annu Rev Public Health. 2019;40:147–166. 30601722 10.1146/annurev-publhealth-040218-044115 PMC6494096

[11] Pedraza FI, Nichols VC, LeBrón AMW. Cautious citizenship: The deterring effect of immigration issue salience on health care use and bureaucratic interactions among Latino US Citizens. J Health Polit Policy Law. 2017;42:925–960. 28663179 10.1215/03616878-3940486

[12] Van Natta M, Burke NJ, Yen IH, et al. Stratified citizenship, stratified health: Examining latinx legal status in the U.S. healthcare safety net. Soc Sci Med. 2019;220:49–55. 30391641 10.1016/j.socscimed.2018.10.024 PMC6546429

[13] Morey BN. Mechanisms by which anti-immigrant stigma exacerbates racial/ethnic health disparities. Am J Public Health. 2018;108: 460–463. 29470116 10.2105/AJPH.2017.304266 PMC5846442

[14] Torres JM, Deardorff J, Gunier RB, et al. Worry about deportation and cardiovascular disease risk factors among adult women: the Center for the Health Assessment of Mothers and Children of Salinas Study. Ann Behav Med. 2018;52:186–193. 29538629 10.1093/abm/kax007 PMC5858722

[15] Krieger N, Huynh M, Li W, et al. Severe sociopolitical stressors and preterm births in New York City: 1 September 2015 to 31 August 2017. J Epidemiol Community Health. 2018;72:1147. 30327451 10.1136/jech-2018-211077 PMC6252370

[16] Losonczy LI, Hsieh D, Wang M, et al. The Highland Health Advocates: a preliminary evaluation of a novel programme addressing the social needs of emergency department patients. Emerg Med J. 2017;34:599–605. 28642372 10.1136/emermed-2015-205662

[17] Tobin Tyler E. Medical-legal partnership in primary care: Moving upstream in the clinic. Am J Lifestyle Med. 2017;13:282–291. 31105492 10.1177/1559827617698417 PMC6506975

[18] Kimball S, Maju M, Singh N, et al. Embedding an Immigration Legal Navigator in a Primary Care Clinic. Ann Fam Med. 2019;17:177. 30858264 10.1370/afm.2360 PMC6411401

[19] League A, Donato KM, Sheth N, et al. A systematic review of medical-legal partnerships serving immigrant communities in the United States. J Immigr Minor Health. 2020. DOI:10.1007/s10903-020-01088-1 PMC7518399 32978741

[20] Saadi A, Ahmed S, Katz MH. Making a case for sanctuary hospitals. JAMA. 2017;318:2079–2080. 29049516 10.1001/jama.2017.15714

[21] Saadi A, Molina US, Franco-Vasquez A, et al. Assessment of perspectives on health care system efforts to mitigate perceived risks among immigrants in the United States: A qualitative study. JAMA Netw Open. 2020;3:e203028. 32301990 10.1001/jamanetworkopen.2020.3028 PMC7165299

[22] Woolhandler S, Himmelstein DU, Ahmed S, et al. Public policy and health in the Trump era. Lancet. 2021;397:705–753. 33581802 10.1016/S0140-6736(20)32545-9

[23] Clínica Msr. Oscar A. Romero. Patient Satisfaction Survey Analysis 2017, Internal Documents.

[24] Clínica Moseñor Oscar A. Romero. Provider Directory. Clínica Romero. Available at https://clinicaromero.com/provider-directory Accessed March 22, 2021.

[25] National Center for Chronic Disease Prevention and Health Promotion. Understanding the Training of Trainers Model. Available at https://www.cdc.gov/healthyschools/tths/train_trainers_model.htm. Accessed October 28, 2021.

[26] Simmer-Beck M, Wellever A, Kelly P. Using registered dental hygienists to promote a school-based approach to dental public health. Am J Public Health. 2017;107(S1):S56–S60. 28661808 10.2105/AJPH.2017.303662 PMC5497873

